# The trends of female sterilization in India: an age period cohort analysis approach

**DOI:** 10.1186/s12905-022-01857-0

**Published:** 2022-07-05

**Authors:** Anjali Bansal, Laxmi Kant Dwivedi, Balhasan Ali

**Affiliations:** Department of Survey Research and Data Analytics, International Institute for Population Sciences, Mumbai, India

**Keywords:** Age-period cohort analysis, Maximum entropy, Female sterilization, DHS, India

## Abstract

In India, sterilisation is the most frequent method of modern contraception, and is primarily used by women. The contemporaneous assessment of sterilisation literature focuses only on trends and patterns that are limited to socioeconomic considerations, ignoring the cohort and period issues. No study has employed Age Period Cohort (APC) analysis to highlight the effect of APC on a particular outcome to yet. We have used maximum entropy method modelling to analyse the individual influence of APC on female sterilisation in India using the four rounds of the National Family Health Survey (NFHS). While the older group had higher sterilisation rates than the younger cohort, the age effects were found to have a standard inverted U-shaped curve, with women sterilising in their mid-30s as the might have completed their desire family size. The analysis found high rural-urban differentials in utilising female sterilisation, highlighting the relevance of education and empowerment in contraceptive decision-making among the educated one. Female sterilisation has become less common among Muslims in India over time, and among uneducated women, and it has shifted to later ages with each succeeding period. This was determined to be concerning in terms of India's future fertility. Since 1947, the government has implemented numerous policies to provide women with a variety of contraceptive options; however, the dominance of female sterilisation throughout all periods demonstrates that the government's efforts to provide temporary methods were futile.

**Supplementary Information:**

The online version contains supplementary material available at 10.1186/s12905-022-01857-0.

## Introduction

In the earlier 1940s, both the developed and developing countries have experienced high fertility rates but with the higher political stability, per capita income and rapid urbanisation, there has been impressive gains in the education and heath indicators which halted theincreasing growth rate in the developed world in the early 1990s [1–3], but developing countries still faces the rapid growth rate [4]. In India, the rapid population growth has been a cause of concern since 1947s after independence among the policymakers. During the 1950s, the policymakers realized that with the limited resources, the country's growing population could dampen economic growth. Therefore, the First Five-year Development Plan (1952–57) recognized the urgency to deal with the problem of family planning and population control, so India launched its first official family planning program in the 1952s. Substantial financial investments were made to expand service delivery points so that contraception can be easily assessable to couples [5]. During the programme, the government made available different contraceptive methods to couples like condoms, Intrauterine contraceptive device (IUD), diaphragm, and sterilization [6]. The contraception were made available at hospitals, health centres and birth- control clinics [4]. Despite the concerted efforts, the birth rate remained more or less the same, 41 per 1000 women in the reproductive ages from 1951 till 1971 [4]. So, the government changed its policy of persuasion, and set a national target of 4.3 million sterilization in a period of 12 month (April 1976 to March 1977), which is almost twice the previous 12 month mark [4]. The mass sterilization was part of the earlier family planning programme but during the emergency period (1974–79) [7, 8], there were many highly unethical instances of sterilization  where mostly men were sterilized unknowingly in hospitals [9]. Civil servants were told if they produce a fourth child after September 1977, they will lose their jobs [10]. The government officials also  fixed some quota, and every state was asked to achieve the goals by any means [8]. The increasing sterilization rates caused a massive political upheaval, and a new government was formed, which again shifted the family planning programme to a family welfare approach, in which couples acquitted with “basket of choices” [11]. In basket of choices couples can decide on which contraception to use to control their family size, which included five official methods- female, male sterilization, IUD, oral contraceptives, and condoms, but in India female sterilization emerged as the only method adopted among all the currently married or in-union women in India[12, 13].

Globally, out of 1.9 billion women of reproductive ages, around 842 million women were using the modern method of contraceptives [14], with 219 million women relying only on female sterilization followed by male condoms (189 million) worldwide [15]. In most regions, the prevalence of female sterilization has declined since 1994, but in Central and Southern Asia, the prevalence of female sterilization increased to 21.8 per cent in 2019 [15]. According to the United Nations data in 2011, India alone is responsible for 36% of female sterilization worldwide [16]. According to the latest data from NFHS-5 (2019–21), in India, around 38% women were using contraceptives which increased to two percentage points since  NFHS-4 (2015–16) [13, 17]. The mean age of female sterilization was 26 years which remained stagnant since the last NFHS  and majority women belonged to the rural part of India who undergo sterilization at earlier ages [13]. In July 2012, the international family planning was held in London, where unprecedented levels of financial and political commitments were made to achieve the goal of 120 million users of family planning by 2020 [18]. India also in 2012, committed to spend $3 billion by 2020 for family planning (FP) program, to drive access, choice and quality of FP services and to increase the modern contraceptive usage from 53.1% to 54.3% and to ensure 74% of the demand for modern contraceptives is satisfied by 2020 [19]. It was ensured that both modern spacing and permanent methods would be promoted, but the share of the spacing method is quite low compared to permanent method.

There is a shred of ample evidence that female sterilization has increased globally and in India. Various studies in India have studied the trends of the contraceptive method by different predictors, and shows that age, period and cohort has an independent effect on female sterilization [20–22], they did not throw light on age-period-cohort characteristics. Age-period-cohort factors have a large influence on sterilisation simultaneously, and suffer due to identification problem. It is possible that at same time age-specific characteristic and changes in economy and technology impact sterilisation adoption as well as cohort characteristics like social norms and educational attainment also affect it. So, without using APC, it is not possible to estimate separate factors effect. This method is generally used by epidemiologists [23–25], economists [26, 27], but now is also used in demography to identify the changes over time, period, different birth cohorts in a particular health outcome [28]. Different age groups are generally exposed to specific changes related to education, parity, and work, while various periods (dates) are mostly exposed to different events such as famines, wars, recessions, pandemics (epidemics), and birth cohort experience different histories, peer socialization [26]. Thus it is essential to disengage the effects of age, period, and birth cohort on a particular health outcome. So, in this study, we have used the maximum entropy approach using APC modelling to examine the individual effect of age, period, and successive birth cohort on female sterilization in India using the four rounds of the National Family Health Survey (NFHS).

## Data Sources

We have used data from the four National Family Health Survey (NFHS) rounds, where first round was conducted in 1992–93, second in 1998–99, third in 2005–06, and fourth in 2015–16. NFHS is a nationally representative cross-sectional survey that includes representatives' samples of the household throughout India. The survey provides estimates at the state, national and district level (only in NFHS-4) on various socio-economic and program dimensions, which are critical for implementing the desired demographic and health parameters changes. A stratified, two-stage sampling method was used in NFHS which is mostly used in all Demographic health surveys all over the countries to obtain a representative sample of households. The 2011 census served as the sampling frame for the selection of Primary Sampling units (PSUs). PSUs are villages in rural areas, and Census Enumeration Block (CEB) in urban areas. In urban areas, CEB information was obtained from the Office of the Registrar and Census Commissioner. In the first stage of sampling, villages were selected from the sampling frame within each rural stratum with Probability Proportional to size (PPS). In the second stage, in every selected rural and urban clusters, 22 households were selected randomly using systematic sampling (the detailed sampling method is available here [13]). NFHS-1 comprises 88,562 households and 89,777 eligible women. While NFHS-2 comprises 92,486 households with 90,303 women in the reproductive age group (15–49). NFHS-3 comprises of 1,09,041 households, 1,24,385 eligible women. Unlike NFHS-3, in NFHS-4, the sample was estimated for district level, with 6,01,509 households with 6,99,686 eligible women.

### Sample

In this study we have only included currently married women of the reproductive ages 15–49 who were currently residing in the household, we have only considered the de-facto women. NFHS-1 comprises 84,289 married women aged 15–49. NFHS-2 comprises 84,862 married women aged 15–49, while NFHS-3 comprises 87,925 married women. NFHS-4 comprises 492,091 currently married women aged 15–49. The overall pooled sample for the analysis comprises of 749,167 currently married women of reproductive ages 15–49.

### Statistical analysis

The dependent variable (Female sterilization status) was computed using the question on the current method used for family planning. The women were categorized as 1 if “sterilized”, 0 "not sterilized". The women who were not using any method or not being sterilized were considered as not sterilized. The data on contraception use was extracted from all four rounds of NFHS and pooled. The individual women's sample weight was adjusted while the data were pooled. Our pooled dataset is not a panel dataset. However, we constructed synthetic cohorts by categorizing individuals using their age-period identifiers and follow them. To identify the birth cohort, the age of women was subtracted from the period of the survey. Each cross-section survey round of NFHS is representative of the population; we can extract the information about the changes in the female sterilization over life-cycle factors.

We then decompose the changes in the female sterilization into age, period, and cohort effects using the synthetic cohort analysis APC methodology. Cohort and age were measured in single-year increments. The major difficulty in estimating the separate impact of age, period, and cohort is the identification problem, which is mainly caused due to the linear relationship among the latter. Different authors have used different approaches to study the APC model, Yang Model [29], Hanoch-Honig/Deaton-Paxson normalization approach [27, 30] and maximum entropy (ME) approach by Browning, Crawford and Knoef [26].

In most of the demographic and epidemiological studies, authors have used Yang model which used the intrinsic method. But this approach also impose some requirements on the geometric orientation of the parameter vector in the parameter space, and is only applicable when we have the data available for many periods [26]. In this study, we have used the ME approach over other approaches because it does not impose any restrictions on identifying the model. The approach is built to provide a framework that can formalize the uncertainty in the model, and instead of providing one unique solution, it estimates the most likely solution [26]. In this approach all the coefficients of the APC model is parametrised in terms of probability distribution over set of possible solutions, and it is the best possible method to apply when the outcome variable is bounded (0,1) [26].

In this study, we first presented the aggregate female sterilization use analysis at national level, and for six states of India, Bihar, Karnataka, Madhya Pradesh, Odisha, Tamil Nadu, and Uttar Pradesh. Then we conducted the separate analysis by place of residence (urban vs rural), wealth index (poor vs non-poor), religion (Hindu, Muslims, and others), parity (1 vs 2+), and level of education (no education, primary, secondary, higher), age at marriage (< 18, 18–24, 25+).

## Results

In India the female sterilization rates varied at each age, birth cohort, and period of the survey. The rates of female sterilization increases after the age of 27. It was found that 30% of the women were getting sterilised after the age of 27. At the age of 36, almost half of the women sterilized in India (Additional file 1: Table S1). Among the birth cohort, women born in the year 1956, were more likely to get sterilized compared to all other women born in different birth cohort. Also, women born after independence (1947) were more prone to get sterilized (Additional file 1: Table S2). The sterilization rates increased since 1992–93 (NFHS-1) in India, which was only 27% in 1992, while in 2015–16 it increased to 36% (Table 1).

**Table 1:** Period wise prevalence of female sterilization among currently married women in India by Place of residence, Wealth quintile, Religion, Parity, and educational status

	Period of the survey				Total
	1992	1998	2005	2015	
Contraception use	27.4	34.1	37.7	36.0	**31.5**
Place of residence					
Urban	33.7	37.8	39.1	36.0	**35.5**
Rural	29.9	35.4	38.5	36.4	**34.8**
Wealth Quintile					
Poorest	23.5	27.2	31.4	29.3	**27.8**
Poorer	25.5	32.0	38.3	35.6	**33.6**
Middle	33.2	41.0	43.0	40.5	**39.2**
Richer	36.3	42.3	43.9	40.0	**39.4**
Richest	35.3	37.3	36.4	35.0	**34.5**
Religion					
Hindu	14.5	36.2	40.2	38.2	**37.8**
Muslim	30.4	19.6	21.6	20.9	**21.0**
Others	29.2	36.4	37.8	39.1	**32.4**
Parity of women					
1	4.3	4.9	6.2	7.8	**6.7**
2 +	42.6	48.5	51.8	49.5	**47.8**
Educational Status					
No education	29.3	35.8	41.4	43.0	**38.2**
Primary	40.0	43.3	45.1	42.9	**41.8**
Secondary	30.9	36.0	35.1	32.9	**32.7**
Higher	16.9	22.6	19.7	19.2	**19.0**
Age at marriage					
< 18	29.0	36.7	42.0	43.4	**40.2**
18–24	24.8	30.7	32.5	31.1	**30.2**
25 +	17.0	18.8	20.7	22.4	**21.8**

### Age effect

**Fig. 1:**
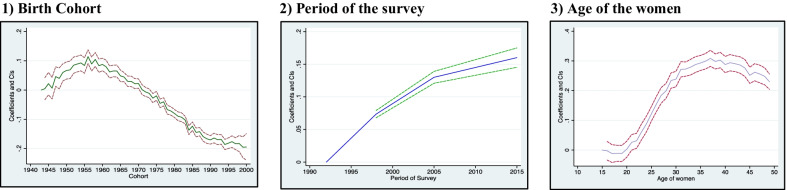
Decomposition Results: Female sterilization in India, NFHS, 1992–2016.

**Fig. 2:**
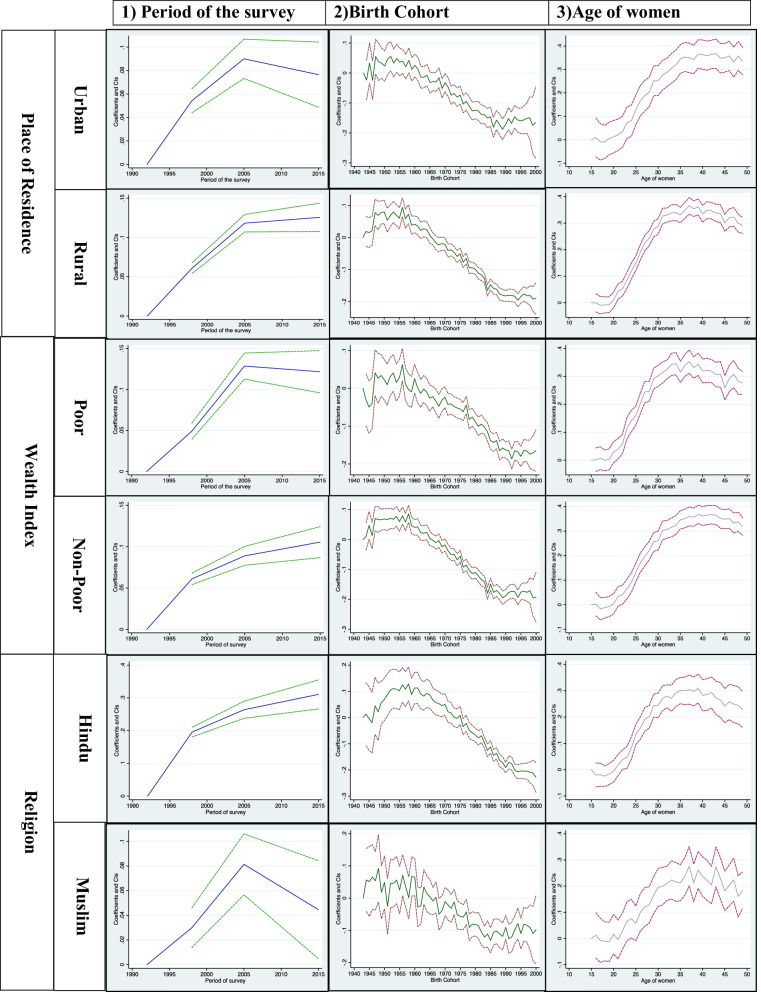

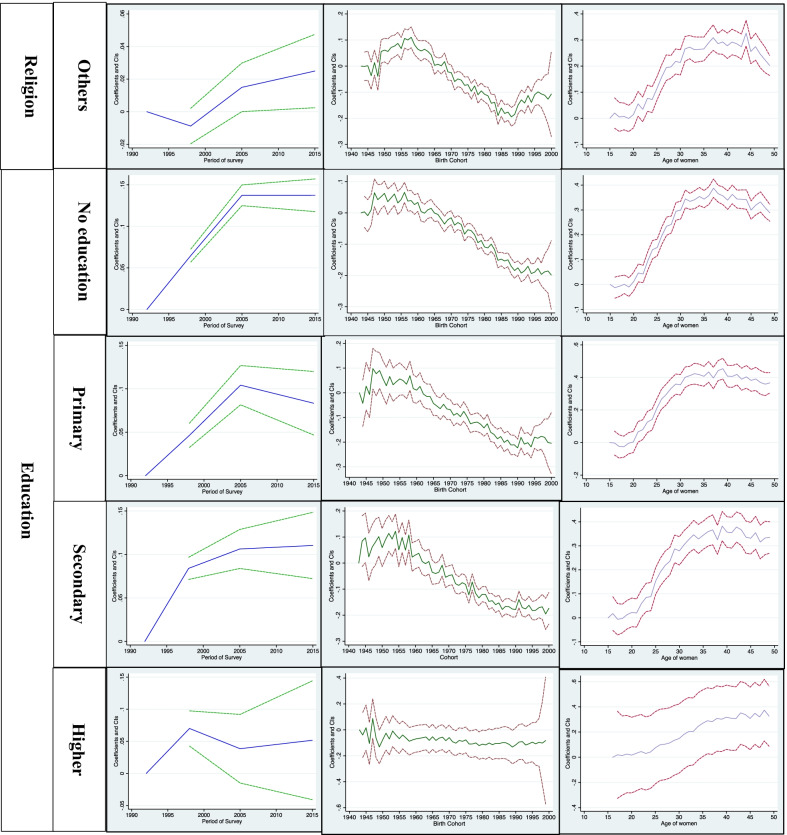

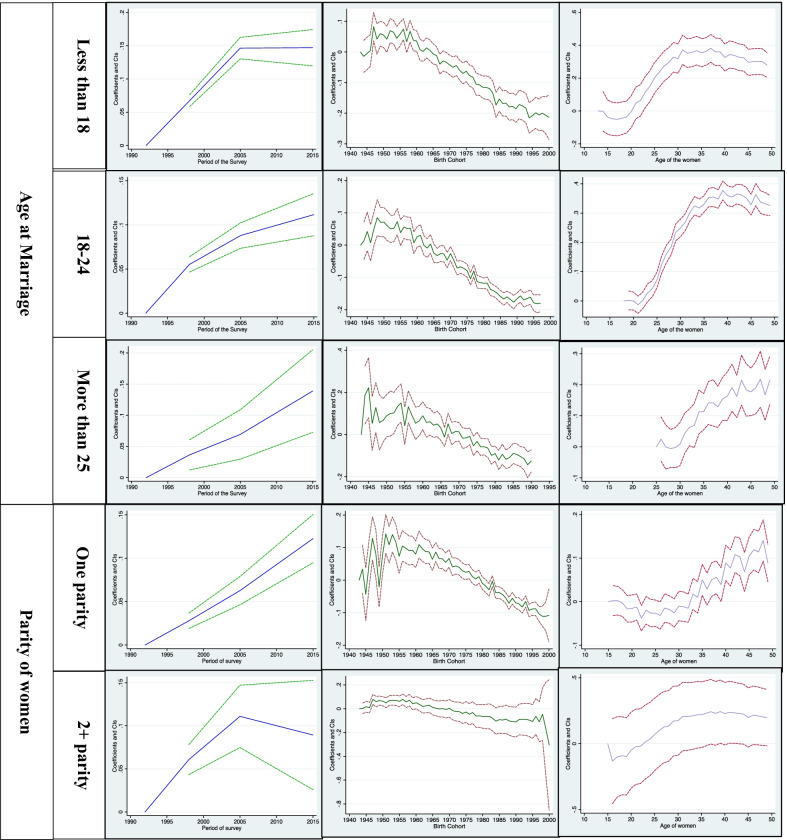
Decomposition Results of Female sterilization for covariates, NFHS, 1992–2015

**Fig. 3:**
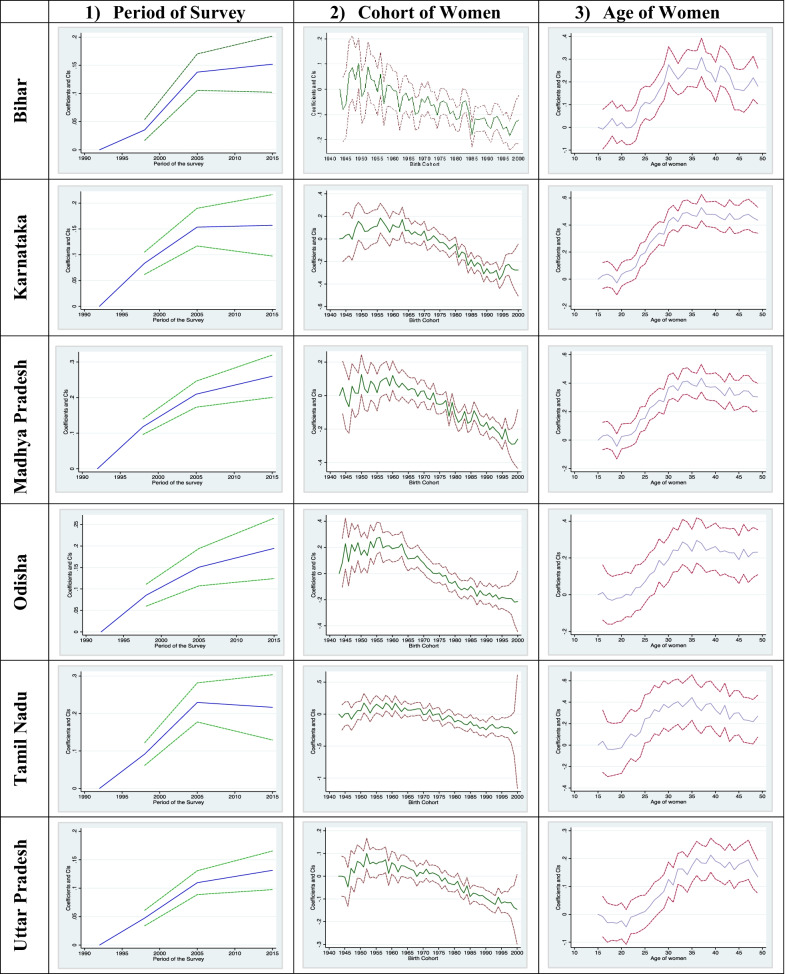
Decomposition Results of Female sterilization for selected states of India, NFHS, 1992–2015

Additional file 1: Table S1 predicts the age-wise distribution of currently married women by residence, wealth quintile, religion, parity of women, and educational status. Figures 1 and 2 shows the estimated age, year, and cohort profiles of India's predicted female sterilization rate and by different covariates. Figure 3 displays the age, year, and cohort profile for the selected six states of India. The Additional file 1: Table S1 presents increasing trends of female sterilization as the age increases. It was found that 39% of the women got sterilized at the age of 30. The predicted probabilities have a standard inverted U-shaped curve, which shows that the sterilization rates were low in the younger ages, increased after age 25 and then remained almost stagnant in the ages 35 to 40 years, and again showed a declining trend in the later ages, 45+.

For all the covariates, the predicted probabilities have an inverted U-shape, except for women with high educational status andwith parity of more than two. The decomposition analysis shows that in urban areas, the peak of female sterilization generally starts from their 40s, while in rural areas, mostly women get sterilised in their 30s. The percentages obtained in Additional file 1: Table S1 also confirm the same, where there exists a rural–urban differentials in sterilization adoption. It was found that around 35% of the urban women and 40% of rural women adopted female sterilization at the age of 30. Around 33% of the rural poor women were sterilized at the age of 30, while among the richer group, around 30% were sterilized at the age of 30. The richest quintile were found delaying their age at sterilization for a minimum of three-year than poorest women (Additional file 1: Table S1). The pattern of sterilization among the poorer, middle and richer quintile were found to be almost similar, where 42% of poorer and richer women get sterilized in their 30s, while 45% of the middle class women get sterilized at the age of 30. The decomposition analysis found that the peak of female sterilization among the poor women starts at age 30, and remains stagnant till age 40, then decreases with age. Among the non-poor women, female sterilization mainly occurs after at the age 40 and remains constant till the end of their reproductive span. The women's religion also depicts the changes in the adoption of female sterilization. It was found that around 38% and 32% of the Hindu women and other women (Sikh, Jain, Parsis, Buddhist) adopt female sterilization, respectively. In contrast, among Muslims, the prevalence was only 21%. While looking at the trend of adopting female sterilization, the Hindu and other religious women mainly adopt female sterilization after attaining age 30. Then the rates remained constant and then declined after the age of 45. While among the Muslims, the rates of sterilization were very fluctuating. Women with higher educational level, the female sterilization rate for all ages were almost similar. Additional file 1: Table S1 shows that women with higher education got sterilized mostly after their 40s, but women with no formal education mostly sterilized in their mid-30s. Women who married before age 24, majority sterilized after the ages 40s. The decomposition analysis also confirms the same, women who married at ages less than 15, they mostly got sterilized in their mid 30s, while those who married at ages more than 25, they mostly got sterilized in their 40s. The sterilization rate by the parity of the women also fluctuates at different ages. For parity one, women may be based on their desired family size got sterilized. It was found women with parity more than two, got sterilized in their mid-30s, and then the rates of sterilization continued to be constant with the increase in the age of women (Fig. 2).

### Period effects

This section will discuss the year effects with the cyclical fluctuations in the female sterilization rates in India. Figure 1 shows the overall year effect in the predicted sterilization rates. Figure 2 shows the year effects by rural/urban residence, wealth index, religion, educational status, and parity of currently married women in India. Table 1 shows the period wise percentage distribution of currently married women for the latter covariates. The female sterilization was only 27% in NFHS-1, which increases drastically in NFHS-2 (34%), and in NFHS-3, the sterilization rates increased by three percentage points (2005–06). But, the latest estimates of NFHS-4 shows a slight decrease in the prevalence of female sterilization (Table 1). For every covariate, we observe a different fluctuating pattern in India. In both rural and urban areas, the sterilization rates were very high in urban areas as compared to rural areas in NFHS-1, but with the rapid urbanisation, the rates of rural and urban areas were almost similar in every subsequent period. Among the poor, the sterilization rates were higher compared to non-poor. The religion also shows fluctuations in the rates of sterilization. Among the Muslims, the rates of sterilization were very less at all periods of NFHS than Hindu and other caste. Among the Hindus, the rates of sterilization increased very rapidly in NFHS-2 and then observed almost the stagnant pattern since NFHS-3 and 4. While in Muslims, the rates of sterilization slightly increased in NFHS-2 compared to NFHS-1, but in NFHS-3, the female sterilization rates increased drastically and then again observed a declining trend in NFHS-4 (2015–16). The contraceptive increase is always associated with the increase in the educational status of women, which enable them to decide their contraceptive choices. The decomposition analysis found that for women with no primary education, the rates of sterilization drastically increased in 2005–06, and then have an almost constant trend. Among the higher educated women, the sterilization rates increased in 1998 and then decreased in NFHS-3, and after that, the predicted probabilities again decreased in NFHS-4. For age at marriage, the mean probability of getting sterilized in NFHS-1 was very small for women married less than 18, but it NFHS-3 it increased drastically, and remained constant in NFHS-4. For women married at the age more than 25, the sterilization are still quite low in NFHS-4, though it has increased since NFHS-1. For parity one, it was observed that for every subsequent NFHS survey, the mean probability of adopting sterilization increases among women. So, there was a high likelihood that women in NFHS-4 (2015–16) were more likely to get sterilized after attaining one parity.

### Cohort effects

Additional file 1: Table S2 predicts the birth cohort wise distribution of currently married women by residence, wealth quintile, religion, parity of women, and educational status. It was found that women born early were more prone to adopt female sterilization than younger women in India. The birth cohort 1956 has the highest number of sterilization users in India compared to all birth cohorts. In the early cohort, more of the urban women were found adopting female sterilization than rural women. Afterwards, the rates of sterilization decreased with subsequent birth cohort. The use of female sterilization by the birth cohort was almost similar for all women, irrespective of their socio-economic background and life cycle factors (Fig. 2). Female sterilization was almost similar among the poor women in the older cohort. Still, among the younger cohort, female sterilization among the poor was more than non-poor (Fig. 2). The pattern of sterilization was high in the older cohort of Hindu women, but after the 1965 cohort, a declining trend was observed in the sterilization rates. There was a fluctuating pattern in the predicted probabilities of sterilization use among the Muslims. For educational status, female sterilization was higher among the older women who have less education than younger women with more education. For higher educated women, the pattern of sterilization use is almost stagnant after the birth cohort of 1960. Older cohort women who were married before 24 years are more likely to adopt female sterilization than younger cohort. Majority women born in 1960, and married before age 18 were found adopting female sterilization. There were visible differences in the sterilization use cohort wise by the parity of women. Among the women with one parity, the use of sterilization peeked in the birth cohort 1945–1950, then again declined, and then followed a declining pattern.

### State-wise decomposition analysis

The female sterilization in all the selected states has shown an increasing trend, except Odisha, whose sterilization prevalence remained the same since NFHS-1 (Additional file 1: Figure S1). For age, all the states followed an inverted U-shaped curve except Bihar, where there were many fluctuations.

In the Southern states of Karnataka, and Tamil Nadu, the prevalence of female sterilization was very high in the initial NFHS-1 (Additional file 1: Figure S1). The mean predicted probabilities show that the use of female sterilization in Karnataka and Tamil Nadu was stagnant after 2005–06 (Fig. 2). In Karnataka, sterilization rates peaked at ages 30, while in Tamil Nadu, it attained the highest value at the age of 25. In Tamil Nadu, the predicted probabilities was higher in all the birth cohorts, while in Karnataka, it showed a declining trend among the younger cohort. In the Eastern region, Bihar had the lowest level of contraception use in India. In Bihar, the sterilization rates peaked at age 30 and remained constant till age 35, and then showed a fluctuating trend. In Odisha, a similar trend was observed, where mostly women aged 30 years got sterilized, and the rates were higher among the older and low among the younger cohorts.

In the central region of India, the rates of sterilization were found to observe an increasing trend with subsequent NFHS survey. In Madhya Pradesh and Uttar Pradesh, women mostly got sterilized at ages 30. The sterilization rates are also higher among the older cohort than the younger ones (Fig. 3).

## Discussion

This study is the first study in family planning that has canvased the APC model and disengaged the effects of age, period, and birth cohort on female sterilization. The results in the line from the previous study have found that education, place of residence, household wealth, and religion are significant contributors to the adoption of female sterilization in India.

Age effect represents the biological and social processes and developmental changes across life [29]. Our study shows that the adoption of female sterilization increases with the increase in the age of women. The decomposition analysis found an inverted U-shaped curve for all the indicators for the age of women expect for women with high educational status and with parity more than two. Mostly women found adopted female sterilization in their late 30s, and then follow a stagnant pattern as the age increases. It was found that the urban women were less prone to adopt early female sterilization, than rural women, which shows the role of education and empowerment in the contraceptive choices among the educated group of the society. Previous studies also in the same lines found that rural women adopt early contraception due to lack of autonomy over their health decisions [21], lack of awareness, and husband's preference over fertility choices [31], son preference [32, 33], and also due to the patriarchal norms that considers vasectomy as a threat to masculinity and sexuality [34]. While in urban areas, women are more empowered and marry at later ages as they first complete their education and establish themselves, which delay their age at childbearing, and thus the age at sterilization. Also, the contraceptive choices among the poor rural women are very minimal, and they generally adopt only female sterilization as their contraceptive method once their desired family size is completed [35, 36]. Similar to our findings, two studies found that the individual's socio-economic background affects their contraceptive choices [37, 38]. Religion of women also has a major role in depicting the contraceptive behaviour of women. Study similar to our finding found that sterilization was higher among Hindus than Muslims as Muslim men generally desire large families than Hindus, hence delaying the age at sterilization [39]. Also, Islam religion encourage child bearing which restricts the use of family planning among them [40]. But with the diffusion of education among them, the rates of sterilization has increased remarkably [41].

Period effect refers to changes in an individual across all age groups in a consistent manner, due to major events like pandemic, or war which affects everyone. The period effect suggests that the use of female sterilization increased from 1992-to 2005 and then remained almost stagnant in the latest period, 2015–16. The increase in women's education over the period may empowered women to adopt spacing method, and also enables them to communicate with their closest social network  about their contraceptive choices [42] and adopt a certain method based on their choices. In rural areas, female sterilization was found to be adopted more among women than urban women. In India, since independence government adopted many policies to control the growth rate which attributes to the increasing rates of female sterilization. India was the first to launch family planning programme in 1950s, which provided married women with different contraceptive choices. After that many policies were timely implemented by government. During 2005–06, India launched remarkable National Rural Health Mission (NRHM) in rural areas, and in later in 2011 in urban areas with the motive to increase the maternal and child care services (MCH) addition to modern contraception prevalence rate (mCPR) in the country [17, 43]. In rural areas with the integration of the NRHM, ASHA workers played a vital role in motivating women to adopt family planning and mainly focused only on female sterilization [43], while in urban areas, the women are more empowered and have more contraceptive choices which enable them to decide their contraceptive method [37, 38], and adopt more of spacing methods. It was found that after 2005–06, the sterilization rates among the poor women showed a declining trend, and in non-poor in remained more or less same. The contraceptive choices of the poor women are very narrowed, and but with the diffusion of education among the poor, the contraceptive rates increased among them [37], and also NRHM, which was launched in 2005–06 to promote the MCH services motivated poor women to adopt sterilization [43]. Among the Muslims as the education increases, their basket of contraceptive choices also increased, which enabled them to adopt spacing methods [41]. Among illiterate couples, the fertility rates have almost declined due to increased awareness [45, 46]. The contraceptive choices among the higher educated women are generally more than uneducated women, as they have tend to have a social network that influences their choices [41]. Mostly in the earlier periods, women moslty adopt family planning after the parity 4 [38] but nowadays most couples prefers small family norm, and prefer only one child, and soon get sterilized after that.

Also, the cohort refers to the changes in an individual born at a particular time. Our study showed that the adoption of female sterilization was high in the older cohort than younger ones, and a similar pattern was observed in all the states, and covariates, except for Tamil Nadu, where it was observed a similar trend of female sterilization in all the birth cohort. With similar studies, newer cohorts generally rely on the spacing methods of family planning as they are more educated and still in the reproductive age groups [47]. Also, education have a major role in the contraception adoption in India. Earlier studies also in a similar line found that female sterilization is less among educated women who have correct knowledge about how to properly use the spacing method to avert an unwanted birth [48, 49]. In southern states, mostly women got married early, completed their desired family size, and later got sterilized [50, 51]. In Uttar Pradesh, primarily urban women rely on the temporary method of contraceptives[52].

## Conclusion

In this study, we attempted to disentangle the effects of APC on female sterilisation in India. Female sterilisation has been the most extensively used contraceptive method in India for decades, according to the data. The period effect has the largest impact for female sterilisation uptake in India. The educational level of women has the most impact on the sterilization rate in India. As women's educational levels increase, the rates of sterilisation decline. Uneducated women's awareness of the importance of family planning increased as a result of programmes like NRHM [46]. The findings also revealed that women with parity one are frequently sterilised at the age of 30, with the prevalence rising in the most recent cycle of NFHS, perhaps leading to sterilisation regret [53]. In 2015, the government launched three modern methods, injectable, centchroman, and progestin only pills [44] to shift from terminal method to spacing method, but the latest data from NFHS-5 shows an increase in the sterilization rates in India, which shows government efforts to provide spacing methods were futile. In India family planning programme promotes only female sterilisation as the exclusive means of contraception. It's critical to switch from a permanent to a spacing strategy to provide users with the basket of choices which can minimise the regret.

## Supplementary Information

**Additional file 1**. **Table S1.** Age wise prevalence of female sterilization among currently married women in India by Place of residence, Wealth quintile, Religion, Parity, educational status and age at marriage. **Table S2.** Cohort wise prevalence of female sterilization among currently married women in India by Place of residence, Wealth quintile, Religion, Parity, educational status, and age at marriage. **Figure S1.** Trends in Female sterilization use by selected states of India, NFHS.

## Data Availability

The datasets analysed during the current study are from National Family Health Survey (NFHS) for India. The data is freely available from the DHS website The DHS Program—India: Standard DHS.
