# Supplementary material for: The trends of female sterilization in India: an age period cohort analysis approach
Source: BMC Womens Health. 2022 Jul 5;22:272. doi: 10.1186/s12905-022-01857-0 (PMC9254500; doi:10.1186/s12905-022-01857-0)
Supplement: Supplementary file 1 — Additional file 1. Table S1. Age wise prevalence of female sterilization among currently married women in India by Place of residence, Wealth quintile, Religion, Parity, educational status and age at marriage. Table S2. Cohort wise prevalence of female sterilization among currently married women in India by Place of residence, Wealth quintile, Religion, Parity, educational status, and age at marriage. Figure S1. Trends in Female sterilization use by selected states of India, NFHS. [file 12905_2022_1857_MOESM1_ESM.docx]

**Table S1: Age wise prevalence of female sterilization among currently married women in India by Place of residence, Wealth quintile, Religion, Parity, educational status and age at marriage**

|  |  | **Place of residence** | | **Wealth Quintile** | | | | | **Religion** | | | | **Parity** | | | **Education** | | | | | **Age at Marriage** | | | |
| --- | --- | --- | --- | --- | --- | --- | --- | --- | --- | --- | --- | --- | --- | --- | --- | --- | --- | --- | --- | --- | --- | --- | --- | --- |
| Age | **Contraception**  **use** | **Urban** | **Rural** | **Poorest** | **Poorer** | **Middle** | **Richer** | **Richest** | **Hindu** | **Muslim** | **Christian** | **Others** | **1** | **2+** | **No education** | | **Primary** | **Secondary** | **Higher** | **<18** | | **19-24** | **>=25** |  |
| 15 | 0.3 | 0.0 | 0.3 | 0.5 | 0.0 | 0.6 | 0.0 | 0.0 | 0.2 | 0.0 | 0.0 | 0.3 | 0.0 | 24.6 | 0.5 | | 0.0 | 0.1 | 0.0 | 0.2 | | 0.0 | 0.0 |  |
| 16 | 0.2 | 0.4 | 0.1 | 0.2 | 0.0 | 0.4 | 0.2 | 0.0 | 0.1 | 0.4 | 0.0 | 0.1 | 0.0 | 8.5 | 0.2 | | 0.3 | 0.1 | 0.0 | 0.2 | | 0.0 | 0.0 |  |
| 17 | 0.5 | 0.7 | 0.5 | 0.2 | 0.6 | 0.8 | 0.9 | 0.0 | 0.5 | 0.0 | 0.0 | 1.0 | 0.0 | 13.0 | 0.7 | | 0.3 | 0.4 | 0.0 | 0.5 | | 0.0 | 0.0 |  |
| 18 | 0.9 | 0.7 | 1.0 | 0.7 | 0.9 | 1.0 | 1.5 | 0.5 | 0.9 | 0.3 | 2.1 | 1.3 | 0.2 | 13.8 | 1.2 | | 1.0 | 0.7 | 0.0 | 1.2 | | 0.0 | 0.0 |  |
| 19 | 2.0 | 2.6 | 1.8 | 1.3 | 1.8 | 2.5 | 2.3 | 2.2 | 2.0 | 0.8 | 4.7 | 2.4 | 0.5 | 16.6 | 2.6 | | 3.2 | 1.4 | 0.0 | 3.1 | | 0.1 | 0.0 |  |
| 20 | 4.0 | 3.7 | 4.1 | 3.1 | 4.3 | 5.7 | 4.2 | 1.4 | 4.2 | 1.7 | 10.1 | 4.6 | 0.5 | 20.6 | 4.8 | | 5.7 | 3.4 | 0.2 | 7.2 | | 0.1 | 0.0 |  |
| 21 | 6.7 | 5.7 | 7.1 | 6.5 | 7.0 | 8.5 | 6.8 | 3.7 | 7.1 | 2.7 | 9.8 | 7.6 | 0.6 | 23.4 | 8.7 | | 9.7 | 5.6 | 1.2 | 12.8 | | 0.9 | 0.0 |  |
| 22 | 9.6 | 8.0 | 10.2 | 8.5 | 9.8 | 12.4 | 11.1 | 4.5 | 10.3 | 4.9 | 14.7 | 9.0 | 0.8 | 25.4 | 10.1 | | 12.9 | 9.8 | 2.1 | 17.2 | | 3.0 | 0.0 |  |
| 23 | 13.7 | 11.4 | 14.5 | 12.9 | 15.2 | 17.6 | 14.4 | 6.6 | 14.3 | 7.3 | 18.8 | 14.4 | 0.9 | 29.4 | 16.0 | | 18.8 | 13.4 | 3.2 | 23.9 | | 5.6 | 0.0 |  |
| 24 | 17.1 | 13.9 | 18.4 | 17.8 | 17.5 | 21.5 | 18.4 | 9.2 | 18.1 | 8.8 | 18.4 | 17.8 | 1.7 | 30.6 | 19.4 | | 23.0 | 17.3 | 4.0 | 27.2 | | 8.9 | 0.0 |  |
| 25 | 20.7 | 16.8 | 22.2 | 18.1 | 23.5 | 25.8 | 22.9 | 11.7 | 22.1 | 10.5 | 23.1 | 21.3 | 1.9 | 33.4 | 22.6 | | 27.2 | 20.8 | 6.1 | 32.1 | | 11.6 | 0.2 |  |
| 26 | 25.1 | 21.3 | 26.9 | 24.6 | 27.0 | 31.8 | 26.7 | 15.6 | 26.4 | 14.6 | 22.6 | 25.7 | 2.7 | 37.0 | 27.8 | | 34.3 | 25.6 | 7.1 | 36.3 | | 16.9 | 1.0 |  |
| 27 | 29.3 | 25.1 | 31.3 | 27.5 | 31.8 | 35.4 | 33.6 | 18.4 | 31.0 | 15.8 | 28.1 | 28.8 | 3.6 | 40.3 | 31.5 | | 38.3 | 30.8 | 10.4 | 39.6 | | 22.2 | 1.5 |  |
| 28 | 32.2 | 27.6 | 34.3 | 28.5 | 34.2 | 40.4 | 35.7 | 22.3 | 34.1 | 16.9 | 31.4 | 33.0 | 5.0 | 42.3 | 33.9 | | 42.4 | 33.0 | 11.5 | 42.4 | | 25.8 | 2.4 |  |
| 29 | 36.6 | 31.9 | 39.2 | 34.2 | 40.1 | 42.8 | 40.7 | 26.4 | 38.2 | 23.0 | 35.8 | 36.0 | 4.8 | 46.2 | 40.1 | | 44.4 | 37.5 | 16.3 | 46.5 | | 30.6 | 5.8 |  |
| 30 | 38.6 | 35.2 | 40.1 | 33.3 | 42.2 | 45.3 | 41.8 | 30.4 | 40.7 | 21.8 | 36.3 | 37.8 | 8.2 | 46.3 | 40.1 | | 46.5 | 38.8 | 17.6 | 47.0 | | 32.5 | 5.9 |  |
| 31 | 41.6 | 36.6 | 44.2 | 38.1 | 43.9 | 50.8 | 44.8 | 31.5 | 43.2 | 25.6 | 36.6 | 42.3 | 8.0 | 48.8 | 44.3 | | 51.9 | 40.9 | 22.4 | 50.2 | | 35.9 | 10.6 |  |
| 32 | 43.5 | 38.6 | 45.9 | 40.3 | 46.8 | 50.3 | 45.3 | 35.9 | 45.1 | 26.8 | 45.1 | 42.9 | 10.1 | 50.1 | 45.7 | | 52.1 | 44.1 | 21.2 | 50.7 | | 39.4 | 10.4 |  |
| 33 | 45.6 | 40.9 | 48.2 | 42.5 | 47.6 | 52.3 | 49.3 | 37.9 | 47.0 | 30.0 | 42.9 | 44.4 | 12.3 | 51.7 | 47.4 | | 55.3 | 46.2 | 23.9 | 52.1 | | 41.6 | 15.0 |  |
| 34 | 48.0 | 45.2 | 49.5 | 41.0 | 49.8 | 56.0 | 52.9 | 40.5 | 49.4 | 31.3 | 45.8 | 47.0 | 13.6 | 53.8 | 48.4 | | 59.3 | 48.3 | 28.3 | 54.0 | | 44.3 | 17.5 |  |
| 35 | 49.0 | 48.0 | 49.4 | 38.6 | 49.4 | 55.0 | 56.0 | 45.9 | 51.1 | 29.8 | 51.9 | 48.3 | 18.6 | 54.1 | 49.4 | | 55.3 | 49.0 | 32.2 | 53.5 | | 46.0 | 21.8 |  |
| 36 | 50.0 | 48.0 | 51.2 | 42.0 | 50.6 | 55.7 | 55.2 | 46.5 | 51.4 | 31.2 | 53.9 | 49.0 | 18.0 | 54.8 | 50.2 | | 58.9 | 50.0 | 33.7 | 53.6 | | 46.9 | 27.7 |  |
| 37 | 51.7 | 48.7 | 53.5 | 43.8 | 52.8 | 57.7 | 55.8 | 48.0 | 52.9 | 34.2 | 55.3 | 48.9 | 18.9 | 56.6 | 53.2 | | 56.6 | 52.0 | 36.7 | 55.5 | | 48.1 | 28.9 |  |
| 38 | 51.9 | 49.0 | 53.4 | 43.4 | 52.5 | 57.6 | 56.1 | 49.2 | 53.9 | 31.2 | 47.4 | 49.0 | 21.4 | 56.3 | 52.0 | | 60.9 | 51.0 | 35.3 | 54.9 | | 49.0 | 28.1 |  |
| 39 | 52.5 | 51.0 | 53.4 | 42.5 | 51.2 | 58.1 | 60.1 | 48.6 | 53.8 | 35.6 | 51.1 | 47.2 | 19.9 | 56.8 | 52.4 | | 62.4 | 53.1 | 32.4 | 55.6 | | 49.4 | 28.0 |  |
| 40 | 53.3 | 53.3 | 53.4 | 41.4 | 52.5 | 59.7 | 60.8 | 51.6 | 55.6 | 32.2 | 51.9 | 47.7 | 26.8 | 57.4 | 52.8 | | 59.3 | 54.4 | 38.3 | 55.0 | | 50.6 | 34.1 |  |
| 41 | 52.9 | 52.6 | 53.0 | 41.7 | 50.8 | 58.0 | 58.2 | 52.8 | 54.4 | 35.0 | 49.3 | 45.3 | 23.5 | 57.0 | 52.1 | | 60.6 | 54.2 | 37.0 | 53.8 | | 50.6 | 34.8 |  |
| 42 | 54.5 | 54.1 | 54.7 | 42.4 | 51.6 | 59.1 | 59.8 | 56.4 | 55.8 | 33.6 | 53.0 | 47.8 | 29.7 | 57.8 | 53.4 | | 60.7 | 56.0 | 42.2 | 54.9 | | 52.1 | 35.2 |  |
| 43 | 54.0 | 54.0 | 54.0 | 39.8 | 51.5 | 58.3 | 61.0 | 54.8 | 54.7 | 38.6 | 52.6 | 45.6 | 30.2 | 57.2 | 53.3 | | 59.0 | 55.6 | 40.5 | 54.2 | | 51.5 | 40.3 |  |
| 44 | 52.9 | 53.3 | 52.7 | 42.0 | 48.1 | 58.8 | 57.7 | 54.1 | 54.2 | 34.8 | 50.1 | 43.5 | 24.4 | 56.8 | 51.5 | | 60.1 | 54.7 | 38.2 | 52.0 | | 51.5 | 35.0 |  |
| 45 | 53.2 | 54.4 | 52.5 | 37.9 | 49.7 | 59.4 | 59.3 | 56.0 | 55.0 | 32.9 | 50.4 | 43.8 | 32.9 | 56.7 | 52.4 | | 59.1 | 53.9 | 39.0 | 52.5 | | 52.0 | 39.3 |  |
| 46 | 53.1 | 53.8 | 52.8 | 40.1 | 49.4 | 57.1 | 60.5 | 54.2 | 54.9 | 32.6 | 41.7 | 40.1 | 33.0 | 56.2 | 52.0 | | 59.8 | 54.6 | 40.8 | 51.2 | | 52.7 | 36.4 |  |
| 47 | 53.8 | 54.6 | 53.3 | 38.2 | 50.7 | 57.1 | 59.4 | 57.3 | 55.8 | 31.4 | 48.5 | 42.7 | 31.3 | 57.0 | 51.3 | | 60.2 | 58.0 | 40.0 | 51.3 | | 54.2 | 40.0 |  |
| 48 | 53.2 | 55.5 | 51.9 | 41.4 | 45.6 | 56.6 | 60.2 | 57.2 | 54.9 | 30.7 | 51.7 | 39.4 | 32.9 | 56.5 | 51.5 | | 55.8 | 56.5 | 47.8 | 50.5 | | 52.6 | 43.4 |  |
| 49 | 52.7 | 52.4 | 52.9 | 39.6 | 50.9 | 56.2 | 57.9 | 54.3 | 54.2 | 29.4 | 50.7 | 37.5 | 30.7 | 56.4 | 51.0 | | 60.1 | 55.1 | 37.6 | 50.4 | | 51.9 | 40.9 |  |
| **Total** | **35.1** | **35.5** | **34.8** | **27.8** | **33.6** | **39.2** | **39.4** | **34.5** | **37.8** | **21.0** | **38.9** | **31.1** | **6.7** | **47.8** | **38.2** | | **41.8** | **32.7** | **19.0** | **40.2** | | **30.7** | **21.8** |  |

**Table S2 : Cohort wise prevalence of female sterilization among currently married women in India by Place of residence, Wealth quintile, Religion, Parity, educational status, and age at marriage**

| **Cohort** |  | **Place of residence** | | **Wealth Quintile** | | | | | **Religion** | | | | **Parity** | | **Education** | | | | **Age at Marriage** | | |
| --- | --- | --- | --- | --- | --- | --- | --- | --- | --- | --- | --- | --- | --- | --- | --- | --- | --- | --- | --- | --- | --- |
|  | **Contraception use** | **Urban** | **Rural** | **Poorest** | **Poorer** | **Middle** | **Richer** | **Richest** | **Hindu** | **Muslim** | **Christian** | **Others** | **1** | **2+** | **No education** | **Primary** | **Secondary** | **Higher** | **<18** | **19-24** | **>=25** |
| 1943 | 38.2 | 47.1 | 35.0 | 29.3 | 33.9 | 35.6 | 39.1 | 49.2 | 17.1 | 32.5 | 21.0 | 27.7 | 5.9 | 41.2 | 35.2 | 49.2 | 44.9 | 27.2 | 26.1 | 29.3 | 15.0 |
| 1944 | 39.0 | 42.0 | 37.9 | 32.7 | 21.0 | 37.3 | 52.4 | 45.3 | 16.1 | 29.3 | 59.8 | 28.6 | 16.0 | 41.4 | 36.2 | 45.5 | 49.5 | 40.4 | 24.0 | 34.8 | 40.4 |
| 1945 | 41.8 | 45.0 | 40.7 | 30.3 | 35.8 | 43.5 | 48.7 | 48.2 | 14.3 | 27.5 | 19.9 | 34.8 | 1.9 | 44.3 | 37.9 | 55.7 | 49.3 | 22.6 | 30.6 | 35.9 | 29.1 |
| 1946 | 40.4 | 40.2 | 40.4 | 28.4 | 32.7 | 42.4 | 48.4 | 44.7 | 16.8 | 41.6 | 42.6 | 30.6 | 15.8 | 43.3 | 37.8 | 50.6 | 44.0 | 34.6 | 29.5 | 32.5 | 14.4 |
| 1947 | 47.7 | 49.3 | 47.1 | 32.6 | 40.9 | 55.1 | 51.8 | 54.3 | 19.6 | 31.2 | 40.4 | 35.7 | 22.0 | 51.3 | 44.5 | 58.2 | 52.0 | 46.9 | 32.6 | 40.1 | 17.0 |
| 1948 | 46.7 | 49.9 | 45.4 | 37.6 | 37.4 | 49.3 | 54.3 | 50.1 | 20.3 | 34.2 | 43.1 | 36.6 | 20.4 | 49.6 | 43.2 | 61.7 | 50.3 | 27.4 | 33.2 | 39.6 | 18.5 |
| 1949 | 49.1 | 50.4 | 48.5 | 35.5 | 42.1 | 46.7 | 54.6 | 57.9 | 38.1 | 35.2 | 44.4 | 40.6 | 9.3 | 52.6 | 44.5 | 60.8 | 59.1 | 33.0 | 38.1 | 42.7 | 34.2 |
| 1950 | 50.5 | 50.8 | 50.4 | 36.7 | 42.8 | 54.2 | 57.6 | 55.4 | 40.8 | 32.6 | 32.4 | 42.5 | 23.0 | 53.2 | 47.3 | 59.9 | 57.1 | 35.2 | 40.1 | 44.6 | 27.1 |
| 1951 | 50.3 | 54.7 | 48.3 | 35.7 | 43.6 | 53.7 | 56.8 | 56.9 | 44.0 | 27.5 | 53.5 | 43.3 | 27.7 | 52.5 | 46.7 | 58.6 | 57.7 | 44.0 | 41.6 | 45.8 | 38.3 |
| 1952 | 52.0 | 55.4 | 50.6 | 40.9 | 44.1 | 52.2 | 63.1 | 56.9 | 44.5 | 34.2 | 42.3 | 45.1 | 19.8 | 55.5 | 47.4 | 64.3 | 61.2 | 39.6 | 43.5 | 47.2 | 24.7 |
| 1953 | 50.9 | 51.5 | 50.7 | 37.6 | 45.0 | 56.5 | 58.7 | 53.1 | 45.4 | 27.7 | 41.6 | 45.8 | 23.4 | 53.9 | 47.4 | 59.8 | 58.4 | 40.6 | 44.8 | 44.6 | 29.2 |
| 1954 | 53.0 | 54.2 | 52.5 | 39.1 | 45.2 | 56.2 | 63.6 | 56.7 | 47.0 | 36.1 | 48.5 | 47.2 | 21.2 | 56.2 | 48.3 | 63.2 | 60.8 | 44.9 | 45.8 | 48.5 | 36.8 |
| 1955 | 52.7 | 53.8 | 52.2 | 41.6 | 46.4 | 59.3 | 60.9 | 53.3 | 48.2 | 36.2 | 43.4 | 47.1 | 18.9 | 56.0 | 49.8 | 61.8 | 57.2 | 39.5 | 47.2 | 47.3 | 33.4 |
| 1956 | 55.1 | 54.2 | 55.5 | 43.7 | 52.6 | 57.0 | 62.0 | 57.4 | 53.6 | 33.7 | 53.8 | 48.2 | 20.7 | 58.1 | 51.8 | 64.5 | 60.6 | 40.4 | 51.1 | 50.3 | 33.3 |
| 1957 | 52.6 | 55.1 | 51.5 | 38.1 | 48.0 | 57.4 | 60.7 | 55.1 | 51.2 | 32.8 | 53.6 | 47.0 | 22.4 | 56.5 | 50.2 | 59.8 | 57.7 | 36.3 | 49.0 | 49.7 | 26.5 |
| 1958 | 52.9 | 53.5 | 52.7 | 39.3 | 43.5 | 62.6 | 61.9 | 54.4 | 52.9 | 30.9 | 47.0 | 47.8 | 19.1 | 56.6 | 48.5 | 66.0 | 62.3 | 29.4 | 51.2 | 49.2 | 25.3 |
| 1959 | 51.4 | 51.6 | 51.3 | 37.6 | 47.5 | 59.8 | 58.2 | 51.3 | 51.4 | 37.0 | 47.0 | 45.6 | 18.4 | 55.0 | 48.6 | 63.1 | 55.4 | 31.4 | 51.0 | 46.4 | 24.2 |
| 1960 | 51.8 | 51.8 | 51.8 | 43.5 | 47.6 | 56.4 | 60.0 | 50.6 | 52.6 | 36.5 | 50.9 | 45.6 | 16.6 | 55.6 | 50.1 | 61.4 | 54.7 | 30.3 | 51.6 | 46.5 | 29.1 |
| 1961 | 51.7 | 51.0 | 52.1 | 41.2 | 45.9 | 59.9 | 58.1 | 51.7 | 54.6 | 30.9 | 49.8 | 45.0 | 17.9 | 55.7 | 48.5 | 61.1 | 58.4 | 30.6 | 50.9 | 48.9 | 25.9 |
| 1962 | 46.3 | 44.4 | 47.1 | 35.1 | 45.6 | 53.4 | 53.2 | 44.5 | 50.1 | 29.2 | 46.0 | 39.1 | 16.3 | 50.4 | 43.7 | 57.3 | 50.2 | 28.8 | 47.1 | 41.2 | 21.7 |
| 1963 | 48.6 | 47.4 | 49.2 | 37.5 | 47.1 | 54.4 | 56.5 | 47.0 | 52.8 | 34.2 | 47.8 | 39.0 | 15.6 | 53.1 | 47.4 | 60.1 | 49.7 | 28.8 | 51.6 | 41.6 | 18.3 |
| 1964 | 45.4 | 44.4 | 45.9 | 35.1 | 41.8 | 53.4 | 55.8 | 41.4 | 51.8 | 34.1 | 35.9 | 35.1 | 12.6 | 51.1 | 43.7 | 56.9 | 48.1 | 23.7 | 48.3 | 39.3 | 19.6 |
| 1965 | 45.6 | 43.0 | 46.7 | 36.5 | 46.8 | 54.2 | 51.2 | 39.7 | 52.7 | 32.0 | 41.7 | 32.1 | 12.6 | 51.3 | 45.6 | 56.2 | 43.9 | 26.5 | 49.4 | 38.0 | 19.6 |
| 1966 | 47.9 | 47.2 | 48.3 | 37.9 | 46.0 | 54.3 | 53.6 | 46.0 | 53.3 | 29.9 | 47.3 | 32.5 | 20.9 | 52.9 | 47.3 | 55.9 | 48.8 | 29.5 | 50.6 | 44.9 | 34.5 |
| 1967 | 47.5 | 49.6 | 46.4 | 36.3 | 43.1 | 51.6 | 54.6 | 49.3 | 54.0 | 30.6 | 45.8 | 28.9 | 20.3 | 53.1 | 46.3 | 52.2 | 49.6 | 37.4 | 49.3 | 45.2 | 40.4 |
| 1968 | 46.0 | 47.7 | 45.1 | 32.8 | 44.2 | 50.3 | 52.4 | 47.1 | 52.8 | 29.9 | 44.7 | 25.3 | 17.5 | 52.2 | 44.3 | 51.6 | 49.6 | 31.7 | 47.8 | 44.1 | 34.5 |
| 1969 | 45.6 | 46.9 | 45.0 | 36.2 | 42.5 | 49.8 | 52.3 | 45.4 | 53.0 | 29.3 | 39.3 | 22.3 | 17.2 | 52.1 | 44.9 | 52.5 | 46.9 | 31.0 | 47.8 | 43.0 | 35.0 |
| 1970 | 47.2 | 49.0 | 46.3 | 33.9 | 45.3 | 53.3 | 53.6 | 47.6 | 53.3 | 31.5 | 46.1 | 20.9 | 20.7 | 53.8 | 47.1 | 53.5 | 46.5 | 31.9 | 49.1 | 45.5 | 37.0 |
| 1971 | 42.3 | 44.2 | 41.3 | 33.8 | 38.7 | 48.1 | 46.8 | 42.3 | 49.3 | 31.0 | 41.4 | 16.5 | 10.9 | 51.3 | 40.9 | 47.4 | 45.4 | 27.8 | 45.0 | 39.8 | 28.9 |
| 1972 | 40.7 | 43.3 | 39.5 | 29.9 | 38.5 | 44.4 | 46.5 | 42.2 | 48.4 | 32.9 | 41.4 | 12.4 | 12.2 | 51.3 | 38.9 | 44.5 | 44.6 | 29.1 | 42.2 | 38.7 | 34.0 |
| 1973 | 43.8 | 46.6 | 42.4 | 33.8 | 41.1 | 48.3 | 47.9 | 46.1 | 49.5 | 29.4 | 46.4 | 15.8 | 13.5 | 53.3 | 42.2 | 48.1 | 46.4 | 33.7 | 45.5 | 41.7 | 32.3 |
| 1974 | 38.6 | 41.8 | 37.0 | 30.5 | 36.0 | 42.5 | 42.8 | 39.6 | 46.0 | 28.4 | 39.2 | 8.8 | 9.9 | 51.0 | 35.3 | 44.6 | 43.6 | 26.6 | 38.3 | 38.9 | 31.2 |
| 1975 | 46.3 | 47.5 | 45.7 | 36.7 | 46.7 | 51.5 | 52.7 | 43.6 | 50.9 | 28.9 | 46.6 | 20.3 | 16.1 | 54.6 | 46.2 | 49.8 | 47.9 | 30.9 | 48.1 | 44.6 | 31.9 |
| 1976 | 40.3 | 42.5 | 39.1 | 32.1 | 39.3 | 44.3 | 46.5 | 37.4 | 44.0 | 28.7 | 44.1 | 16.0 | 9.1 | 51.8 | 39.2 | 46.4 | 43.0 | 23.1 | 42.6 | 38.8 | 25.4 |
| 1977 | 43.2 | 41.4 | 44.0 | 36.5 | 44.5 | 48.1 | 45.7 | 40.5 | 46.6 | 25.3 | 42.3 | 27.0 | 11.9 | 52.6 | 42.7 | 50.0 | 44.4 | 25.9 | 46.5 | 41.1 | 23.4 |
| 1978 | 40.2 | 39.7 | 40.5 | 33.7 | 41.2 | 44.3 | 43.1 | 38.1 | 42.1 | 23.6 | 44.4 | 33.0 | 9.2 | 51.3 | 39.1 | 45.1 | 42.4 | 29.0 | 42.9 | 37.0 | 25.7 |
| 1979 | 40.4 | 40.3 | 40.5 | 34.5 | 40.2 | 44.8 | 44.3 | 37.6 | 42.1 | 25.1 | 45.6 | 40.0 | 9.3 | 51.0 | 40.2 | 47.9 | 40.8 | 27.0 | 42.9 | 38.7 | 25.6 |
| 1980 | 41.6 | 41.9 | 41.5 | 33.8 | 41.6 | 46.7 | 46.8 | 39.2 | 44.0 | 24.2 | 46.9 | 44.1 | 11.4 | 51.2 | 41.4 | 47.0 | 42.5 | 27.3 | 45.3 | 39.6 | 21.0 |
| 1981 | 38.5 | 38.0 | 38.7 | 32.6 | 40.1 | 44.6 | 42.3 | 32.1 | 39.9 | 25.2 | 43.1 | 40.9 | 7.7 | 50.3 | 37.6 | 47.4 | 39.5 | 24.7 | 41.5 | 37.5 | 17.4 |
| 1982 | 36.5 | 34.4 | 37.7 | 34.7 | 38.7 | 40.9 | 38.1 | 30.7 | 38.4 | 22.5 | 37.7 | 33.9 | 6.7 | 48.4 | 37.0 | 42.8 | 37.8 | 21.6 | 40.4 | 34.8 | 15.4 |
| 1983 | 36.5 | 32.7 | 38.4 | 34.6 | 39.7 | 41.9 | 37.2 | 29.4 | 38.4 | 21.0 | 42.2 | 33.8 | 7.3 | 47.1 | 37.3 | 42.5 | 38.0 | 19.6 | 42.5 | 33.8 | 9.7 |
| 1984 | 33.9 | 31.0 | 35.5 | 32.4 | 36.9 | 40.3 | 35.5 | 25.0 | 35.8 | 20.4 | 34.4 | 30.1 | 4.8 | 45.9 | 36.0 | 42.0 | 33.2 | 21.9 | 41.3 | 30.0 | 11.1 |
| 1985 | 33.5 | 31.6 | 34.4 | 29.5 | 36.0 | 39.1 | 35.7 | 26.3 | 35.7 | 18.6 | 35.4 | 30.6 | 5.8 | 44.4 | 34.7 | 39.2 | 34.0 | 16.7 | 41.3 | 28.8 | 6.0 |
| 1986 | 30.5 | 27.3 | 32.3 | 27.1 | 33.6 | 35.9 | 34.0 | 22.0 | 32.1 | 19.2 | 33.5 | 27.8 | 3.0 | 45.1 | 32.5 | 34.4 | 32.3 | 16.7 | 37.9 | 27.7 | 6.6 |
| 1987 | 27.5 | 24.2 | 29.0 | 24.3 | 29.0 | 34.5 | 29.6 | 19.2 | 29.7 | 13.8 | 27.6 | 26.1 | 3.8 | 41.1 | 28.1 | 34.2 | 29.3 | 11.0 | 35.2 | 23.9 | 2.5 |
| 1988 | 25.5 | 22.2 | 27.2 | 24.3 | 27.6 | 30.7 | 29.7 | 15.1 | 27.3 | 14.1 | 26.6 | 21.2 | 2.8 | 39.4 | 26.5 | 32.1 | 28.3 | 9.7 | 33.9 | 21.4 | 1.7 |
| 1989 | 21.9 | 18.6 | 23.5 | 22.5 | 23.9 | 27.6 | 22.6 | 12.5 | 23.4 | 12.9 | 21.6 | 17.0 | 2.3 | 36.0 | 23.4 | 28.1 | 24.2 | 6.1 | 32.6 | 15.9 | 1.3 |
| 1990 | 18.1 | 14.4 | 19.7 | 15.7 | 22.1 | 23.5 | 19.0 | 8.6 | 19.8 | 9.2 | 23.6 | 14.3 | 1.5 | 32.0 | 19.8 | 22.8 | 19.4 | 5.6 | 30.2 | 10.7 | 0.1 |
| 1991 | 15.3 | 12.1 | 16.7 | 16.5 | 16.8 | 18.9 | 16.1 | 7.0 | 16.9 | 6.9 | 19.7 | 9.8 | 1.6 | 30.5 | 16.6 | 20.1 | 16.7 | 4.3 | 27.4 | 8.4 | NA |
| 1992 | 12.0 | 10.1 | 12.8 | 11.2 | 13.0 | 16.3 | 12.6 | 5.1 | 12.9 | 6.3 | 18.9 | 8.9 | 0.7 | 30.4 | 13.4 | 16.1 | 12.9 | 3.4 | 24.4 | 5.4 | NA |
| 1993 | 8.2 | 7.4 | 8.5 | 7.1 | 8.3 | 10.3 | 9.8 | 4.2 | 9.0 | 4.1 | 16.0 | 5.3 | 0.6 | 26.2 | 7.4 | 10.6 | 9.4 | 1.8 | 16.8 | 2.9 | NA |
| 1994 | 5.2 | 4.7 | 5.3 | 4.8 | 5.9 | 6.4 | 4.8 | 2.6 | 5.7 | 2.4 | 6.8 | 2.0 | 0.3 | 24.7 | 6.4 | 7.2 | 5.1 | 1.2 | 11.8 | 0.7 | NA |
| 1995 | 2.7 | 2.3 | 2.9 | 2.0 | 2.6 | 4.4 | 2.8 | 0.7 | 2.9 | 1.2 | 13.1 | 2.4 | 0.5 | 21.2 | 2.8 | 4.5 | 2.6 | 0.3 | 5.7 | 0.1 | NA |
| 1996 | 1.5 | 2.6 | 1.3 | 0.9 | 1.1 | 2.1 | 1.6 | 3.1 | 1.5 | 0.9 | 7.0 | 1.4 | 0.5 | 23.3 | 1.8 | 2.9 | 1.3 | 0.0 | 2.8 | 0.0 | NA |
| 1997 | 0.5 | 0.4 | 0.5 | 0.4 | 0.4 | 0.7 | 0.5 | 0.0 | 0.5 | 0.2 | 4.2 | 1.0 | 0.1 | 15.9 | 0.3 | 0.7 | 0.5 | 0.0 | 0.6 | 0.0 | NA |
| 1998 | 0.4 | 0.7 | 0.3 | 0.2 | 0.1 | 0.7 | 1.0 | 0.0 | 0.5 | 0.0 | 0.0 | 0.0 | 0.0 | 21.2 | 0.5 | 0.0 | 0.4 | 0.0 | 0.4 | NA | NA |
| 1999 | 0.0 | 0.0 | 0.0 | 0.0 | 0.0 | 0.0 | 0.0 | 0.0 | 0.0 | 0.0 | 0.0 | 0.0 | 0.0 | 0.0 | 0.0 | 0.0 | 0.0 | 0.0 | 0.0 | NA | NA |
| 2000 | 0.2 | 0.0 | 0.3 | 0.7 | 0.0 | 0.0 | 0.0 | 0.0 | 0.2 | 0.0 | 0.0 | 0.0 | 0.0 | 0.0 | 0.0 | 0.0 | 0.3 | 0.0 | 0.2 | NA | NA |

**Figure S1 : Trends in Female sterilization use by selected states of India, NFHS**
